# Evaluating healthcare services at MahaKumbh 2025, India: A mixed-methods study of visitor perceptions and public health strategies

**DOI:** 10.6026/973206300210789

**Published:** 2025-04-30

**Authors:** Rahul Srivastava, Sidhant Kumar, Prakash Chandra Sidhant, Priyanka Kumari, Dhirendra Kumar Singh, Jyothi Dundappa

**Affiliations:** 1 Department of Oral Medicine and Radiology, Rama Dental College Hospital and Research Centre, Mandhana, Kanpur, Uttar Pradesh, India; 2 Department of Dentistry, Nalanda Medical College Hospital Agamkuan Patna, Bihar, India; 3 Department of Prosthodontics & Crown and Bridge, Nalanda medical College and hospital,Patna, Bihar, India; 4 Department of Periodontology, Kalinga Institute of Dental Sciences, KIIT Deemed to be University, Bhubaneswar, Odisha, India

**Keywords:** Maha Kumbh 2025, healthcare services, mass gatherings, visitor satisfaction, public health, Uttarpradesh government

## Abstract

Maha Kumbh 2025 in India posed major public health challenges due to the high number of pilgrims gathering at a location. Hence, participants from Rama University, Kanpur, gathered visitor feedback on healthcare services during the event. A descriptive survey was conducted with 1000 attendees using a mixed-methods design. Quantitative data were analyzed using chi-square tests and logistic regression. High satisfaction scores were reported for service availability and medical staff competence. Cleanliness and hygiene received relatively lower ratings, indicating areas for improvement. Healthcare services were largely effective due to strong government and institutional efforts.

## Background:

The MahaKumbh Mela, held once every twelve years, is one of the largest religious gatherings globally, attracting millions of devotees, saints and tourists from across India and beyond. The MahaKumbh 2025 in Prayagraj, Uttar Pradesh, witnessed an unprecedented number of visitors, posing significant challenges for healthcare service providers. Ensuring effective healthcare services during such a massive event was crucial for preventing disease outbreaks, managing medical emergencies and maintaining overall public health. Given the large-scale movement of people, temporary settlements and increased risk of communicable diseases, a well-coordinated healthcare infrastructure remained essential [1]. The healthcare system at MahaKumbh 2025 included temporary hospitals, mobile medical units, first-aid stations, emergency response teams and sanitation facilities to cater to the diverse health needs of attendees. Government agencies, non-governmental organizations (NGOs) and private healthcare providers collaborated to ensure seamless medical services. Despite significant planning and resource allocation, past KumbhMelas, including the 2025 event, reported challenges such as overcrowding, inadequate medical personnel, insufficient sanitation facilities and increased risks of waterborne and airborne diseases [2]. Additionally, the strain on local hospitals and emergency services required a well-structured response mechanism to handle both routine medical issues and critical health crises. Visitor perceptions and satisfaction played a pivotal role in evaluating the effectiveness of healthcare services at MahaKumbh 2025. Satisfaction levels were influenced by factors such as accessibility of medical aid, quality of healthcare services, availability of medicines, response time of emergency teams and overall hygiene conditions [3, 4]. The Maha Kumbh 2025 integrates spiritual celebration with world-class healthcare infrastructure, ensuring the safety of over 150 million pilgrims through advanced diagnostics, mobile units, and 24x7 emergency services.

State and private healthcare collaborations, including Ayush integration, exemplify a holistic and scalable model for mass gathering health management [5]. A positive healthcare experience enhanced trust in the system and ensured the safety and well-being of pilgrims, while dissatisfaction highlighted gaps requiring immediate intervention. Therefore, it is of interest to analyze visitor perceptions and satisfaction with healthcare services at MahaKumbh 2025, identifying strengths and areas for improvement. By assessing medical preparedness, disease surveillance, emergency response efficiency and sanitation management, the study will provide insights into the overall effectiveness of healthcare measures implemented during the event. The findings will contribute to policy recommendations for future mass gatherings, helping authorities improve healthcare preparedness and crisis management strategies to ensure a safer and healthier experience for all attendees.

## Materials and Methods:

The study was conducted at Rama University, Kanpur and a multidisciplinary educational institution that actively engaged in providing healthcare, research and community outreach services. Given its strategic location and academic infrastructure, Rama University served as an ideal setting to assess the perceptions and satisfaction of visitors regarding the healthcare services at MahaKumbh 2025. The university hosted individuals who had attended the event, including students, faculty, staff and the general public visiting its premises. Ethical approval was obtained from the Institutional Ethics Committee (RDCHRC/ETHICSCOMMITTEE/2025/0230 dated 202/02/2025), with written informed consent collected from all participants prior to data collection; confidentiality and anonymity were maintained by not recording personal identifiers. A cross-sectional descriptive survey was conducted to evaluate the effectiveness of healthcare services at MahaKumbh 2025 based on visitor perceptions. A mixed-methods approach was used, incorporating both quantitative and qualitative data collection methods to ensure a comprehensive evaluation.

Considering a 5% non-response rate, the final sample size was set at 1000 participants. The study included pilgrims and visitors of MahaKumbh 2025 who had sought medical assistance at Rama University Hospital. A convenience sampling technique was used to recruit participants from different sections of the hospital. Present study includes the individuals who had attended MahaKumbh 2025 and adults aged 18 years and above who were willing to participate in the study. Individuals who had not attended MahaKumbh 2025, those who declined participation and individuals below 18 years of age were excluded from the study.A convenience sampling technique was used to recruit participants from different areas of Rama University, Kanpur, including academic departments, university hostels, administrative blocks and public attendees who had participated in MahaKumbh 2025.A bilingual questionnaire (Hindi and English) was used to collect data on demographics (age, gender, educational background and occupation), experience at MahaKumbh 2025 (duration of stay, health-related issues faced and healthcare services availed), perceptions of healthcare services (availability, accessibility, hygiene, medical staff responsiveness and overall satisfaction) and feedback and suggestions (areas for improvement in healthcare facilities at future Maha Kumbh events). Responses were recorded using a 5-point Likert scale (1 = Very Dissatisfied, 5 = Very Satisfied). Semi-structured face-to-face interviews were conducted with students and faculty members who had attended MahaKumbh 2025, as well as university visitors and staff, to assess their healthcare experiences. Quantitative data from structured questionnaires were entered into SPSS (version 26.0) for descriptive statistics, chi-square tests and binary logistic regression to identify key factors influencing satisfaction, while qualitative data from transcribed interviews were analyzed using thematic analysis to uncover key themes related to healthcare service effectiveness and accessibility.

## Results:

A total of 1000 participants from Rama University who attended MahaKumbh 2025 completed the survey. The results have been analyzed using descriptive statistics, chi-square tests and binary logistic regression to understand the factors influencing visitor satisfaction with healthcare services. Table 1 summarizes the demographic characteristics of the study population. The largest age group was 18-25 years (35%), reflecting the high proportion of students. Gender distribution was nearly equal with 52% male and 48% female participants. The majority of respondents was students (60%), followed by faculty and staff (15% each), while 10% represented the general public. Table 2 displays the mean ratings and distribution of responses on a 5-point Likert scale for key aspects of healthcare service delivery. The availability of services and medical staff competence received high ratings (mean scores >4.0), suggesting that most visitors found these areas satisfactory. The slightly lower score for cleanliness and hygiene suggests there might be opportunities for improvement in maintaining optimal standards. Table 3 summarizes the chi-square tests used to assess whether overall satisfaction was influenced by demographic factors. The results indicate that the role at the university (students, faculty, staff, general public) was significantly associated with satisfaction levels (p = 0.02), whereas age group and gender did not show a significant impact. Table 4 presents the results of the binary logistic regression analysis, which identifies the key predictors of overall satisfaction. Significant predictors include accessibility, cleanliness and hygiene, medical staff competence and availability of services. These results suggest that improvements in these areas are likely to increase overall satisfaction. Although being a student showed a positive trend (OR = 1.20), it did not reach statistical significance (p = 0.08).

## Discussion:

Present study evaluated the perceptions and satisfaction of individuals regarding the healthcare services provided during MahaKumbh 2025. The findings revealed high overall satisfaction, particularly in terms of service availability and the competence of medical staff, while aspects such as cleanliness and hygiene received relatively lower ratings. These results are consistent with previous studies conducted at similar mass gatherings. Studies conducted at previous KumbhMelas and other large-scale religious events, such as the Hajj pilgrimage and ArdhKumbh, have reported similar healthcare challenges and successes. Similarly, a study on the Hajj pilgrimage (Shafi *et al.* 2008) found that despite the presence of advanced healthcare facilities, maintaining hygiene standards and preventing disease outbreaks posed significant challenges [6]. A distinctive aspect of the MahaKumbh 2025 healthcare strategy was the active involvement of the Uttar Pradesh government, which enhanced healthcare delivery by deploying temporary clinics and mobile health units to serve remote and crowded areas, employing real-time monitoring systems and rapid-response teams for disease surveillance and emergencies, forging public-private partnerships with academic institutions to bolster capacity through resource sharing and joint training programs and investing in logistical support such as improved transportation networks and crowd management systems. These initiatives, which mirror strategies noted in previous research Zafeirakis *et al.* 2020, underscore the proactive stance of the Uttar Pradesh government [3]. The integrated approach, involving both governmental and academic institutions, appears to be a critical factor in achieving high satisfaction levels, particularly in terms of service accessibility and staff competence. Based on the study's findings; several recommendations can be made to enhance healthcare delivery during large-scale events. Strengthening hygiene and sanitation protocols by implementing stricter cleaning measures, increasing the availability of hand sanitizing stations and ensuring effective waste management practices is essential. Institutionalizing effective public-private partnerships can create more robust and responsive healthcare systems, leveraging the strengths of both governmental agencies and private entities. Integrating advanced technologies such as telemedicine, mobile health applications and electronic health records will streamline service delivery by enabling real-time data collection and rapid decision-making. Additionally, continued investments in healthcare and transportation infrastructure are crucial for managing large crowds, while regular capacity building and training for healthcare providers on mass gathering management and emergency response will help maintain high standards of service quality [7]. Future research should build upon the insights gained from this study by exploring multiple avenues. Longitudinal studies are needed to examine the long-term health outcomes of individuals who received care during the event, providing a deeper understanding of the sustained impacts of the interventions. Comparative analyses of healthcare delivery models across different regions or between public and private sectors can help identify best practices and inform effective resource allocation. Additionally, evaluating the effectiveness of innovative technological approaches, such as telemedicine and AI-driven health monitoring, will be crucial for optimizing future healthcare responses. Finally, cost-effectiveness studies are essential to analyze the economic aspects of various healthcare interventions during mass gatherings, ultimately assisting policymakers in designing more efficient and sustainable health strategies.

## Conclusion:

The healthcare services provided during MahaKumbh 2025 were generally well-received by visitors, with high satisfaction levels particularly noted in the areas of service availability and medical staff competence. Despite these positive outcomes, lower ratings in cleanliness and hygiene highlight areas that require targeted improvement. The proactive initiatives of the Uttar Pradesh government, in collaboration with various stakeholders, played a pivotal role in ensuring effective healthcare delivery during this large-scale event.

## Figures and Tables

**Table 1:** Demographic characteristics of participants

**Variable**	**Category**	**Frequency (n)**	**Percentage (%)**
Age Group	18-25	350	35
	26-35	300	30
	36-45	200	20
	46-55	100	10
	>55	50	5
Gender	Male	520	52
	Female	480	48
Role at University	Student	600	60
	Faculty	150	15
	Staff	150	15
	General Public	100	10

**Table 2:** Healthcare service ratings

**Service Aspect**	**Mean Score**	**Standard Deviation**	**Distribution of Ratings (1-5 Scale)**
Availability of Services	4.2	0.7	1: 5%, 2: 8%, 3: 15%, 4: 30%, 5: 42%
Accessibility	4	0.8	1: 6%, 2: 10%, 3: 18%, 4: 35%, 5: 31%
Cleanliness and Hygiene	3.8	0.9	1: 8%, 2: 12%, 3: 20%, 4: 30%, 5: 30%
Medical Staff Competence	4.3	0.6	1: 3%, 2: 5%, 3: 12%, 4: 30%, 5: 50%
Medical Staff Competence	4.3	0.6	1: 3%, 2: 5%, 3: 12%, 4: 30%, 5: 50%

**Table 3:** Association between demographic variables and overall satisfaction (Chi-square Test)

**Variable**	**Chi-square Value**	**p-value**	**Interpretation**
Age Group	8.56	0.07	No significant association (p > 0.05)
Gender	4.32	0.23	No significant association (p > 0.05)
Role at University	12.45	0.02	Significant association (p < 0.05)

**Table 4:** Predictors of overall satisfaction (Binary Logistic Regression Analysis)

**Variable**	**Beta Coefficient**	**Odds Ratio (OR)**	**95% Confidence Interval**	**p-value**
Accessibility	0.35	1.42	1.15 - 1.75	0.001
Cleanliness and Hygiene	0.28	1.32	1.08 - 1.62	0.005
Medical Staff Competence	0.4	1.5	1.20 - 1.88	<0.001
Availability of Services	0.22	1.25	1.02 - 1.53	0.03
Role at University (Student vs. Others)	0.18	1.2	0.98 - 1.47	0.08
